# *Listeria* Occurrence in Poultry Flocks: Detection and Potential Implications

**DOI:** 10.3389/fvets.2017.00125

**Published:** 2017-08-11

**Authors:** Michael J. Rothrock, Morgan L. Davis, Aude Locatelli, Aaron Bodie, Tori G. McIntosh, Janet R. Donaldson, Steven C. Ricke

**Affiliations:** ^1^USDA-ARS, U.S. National Poultry Research Center, Egg Safety and Quality Research Unit, Athens, GA, United States; ^2^Center for Food Safety, Food Science Department, University of Arkansas, Fayetteville, AR, United States; ^3^Department of Biological Sciences, University of Southern Mississippi, Hattiesburg, MS, United States

**Keywords:** *Listeria*, poultry, live production, isolation, detection

## Abstract

Foodborne pathogens such as *Salmonella, Campylobacter, Escherichia coli*, and *Listeria* are a major concern within the food industry due to their pathogenic potential to cause infection. Of these, *Listeria monocytogenes*, possesses a high mortality rate (approximately 20%) and is considered one of the most dangerous foodborne pathogens. Although the usual reservoirs for *Listeria* transmission have been extensively studied, little is known about the relationship between *Listeria* and live poultry production. Sporadic and isolated cases of listeriosis have been attributed to poultry production and *Listeria* spp. have been isolated from all stages of poultry production and processing. Farm studies suggest that live birds may be an important vector and contributor to contamination of the processing environment and transmission of *Listeria* to consumers. Therefore, the purpose of this review is to highlight the occurrence, incidence, and potential systemic interactions of *Listeria* spp. with poultry.

## Introduction

Microorganisms such as *Salmonella, Campylobacter*, and *Listeria* represent a considerable concern within the food industry due to their pathogenic properties and their potential to establish infections in humans. It is imperative that intervention strategies be established to reduce risk of foodborne illness to consumers, specifically *Listeria monocytogenes*. However, little is known about the prevalence of *Listeria* spp. throughout the poultry production and processing continuum. *Listeria* species are Gram-positive, non-spore forming, rod-shaped bacteria that are naturally found in the environment, including soil, sewage, feces from animals and birds, and surface water (1, 2, 3, 4). *Listeria* are persistent facultative anaerobes that ideally proliferate in temperatures of 30 to 37°C, but can withstand temperatures between 0 and 43°C (4, 5). In addition to being able to survive a wide range of temperatures, *Listeria* spp. can grow in a variety of salt concentrations, high osmotic pressure, and low pH environments, but succumb to pasteurization (6). There are at least six known species: *Listeria grayi, Listeria seeligeri, Listeria welshimeri, Listeria ivanovii, Listeria innocua*, and *L. monocytogenes*. Of the six species, *L. ivanovii* is pathogenic to animals and *L. monocytogenes* is the only species pathogenic to humans. While *Listeria* commonly colonize multiple mammalian hosts, it remains unclear the exact relationship of *Listeria* with avian species. While a frequent contaminant of ready to eat meats, the relationship with the live bird production aspect is much less clear. In this review, the occurrence, incidence, and potential systemic interaction of *Listeria* spp. with chickens and other avian species will be discussed.

## *Listeria* and Foodborne Disease

*Listeria monocytogenes* can be subdivided into different phylogenic evolutionary lineages (I, II, III, and IV) based on ecology, genomic content, and recombination rates (7). Phylogenetic serotypes are based on cell wall antigen expression and thus can be used to identify variations among all 13 serotypes (7). Lineage I consists of serotypes 1/2b, 3b, 3c, and 4b strains, while lineage II includes serotypes 1/2a, 1/2c, and 3a (8, 9). Phylogenetic evidence has indicated that rare serotypes may have evolved recently, or multiple times, from one of the major serotypes (10). Lineage III belongs to two groups, formerly lineages IIIA/C and IIIB/C known as lineages III and IV, with serotypes 4a and 4c strains (11). However, limited knowledge exists for lineage IV due to rarity and low strain variability, which contributes to seven unclear serotypes. Similarly, little is known about the lineage status of serotype 7 due to lack of availability of such strains (10, 12, 13).

Serotypes that are most commonly associated with human cases are 11 (1/2b) in chicken meat, C1-056 (1/2a) in human, sporadic case, N1-225 (4b) causing human epidemic, and N1-227 (4b) (14). Variations in strains of *L. monocytogenes* are characterized according to their physiological properties and phenotypic characteristics, such as growth behavior, acid tolerance, and resistance to various stresses (14). There has also been identification of strain variance due to heat resistance (14), which could be directly related to those involved in food contamination that carries over to the consumer due to resistance to heat treatments.

Previous reports have shown an increase in the prevalence of *L. monocytogenes* in ready-to-eat (RTE), vacuum packaged, sliced meat products where 95% of all *L. monocytogenes* belonged to Lineage II, serotype 1/2a, with the remaining 5% varying between serotypes 1/2b, 3b, and 4b (15). Kramarenko et al. (16) reported that 93% of all *L. monocytogenes* isolates obtained from meat products belonged to serotype 1/2a and 1/2c (16). Therefore, this suggests that variations between stress and exposure influence which lineage, serotype, and strain is ultimately responsible for contamination.

*Listeria monocytogenes* is the causative agent of listeriosis, where those considered to be most susceptible include the elderly, immunocompromised, and pregnant women (1, 13). Although one of the less common foodborne illnesses, it frequently requires hospitalization (17–20) and has a mortality rate of 20 to 30% (6). The Centers for Disease Control and Prevention Morbidity and Mortality Weekly Report states that of the 123 cases that occurred in 2013, 91% of reported cases resulted in hospitalization (21). There are a variety of symptoms that may arise upon infection, including septicemia, meningitis, and gastroenteritis (22). In pregnant women, it may cause spontaneous abortion, premature labor, and neonatal disease (23). Serotypes 1/2a (lineage II) and 1/2b and 4b (lineage I) are responsible for a majority of the *L. monocytogenes* hospitalized cases (10, 12, 13). Lineage I is responsible for cases among outbreaks of human clinical listeriosis (10, 13), while Lineage II strains exhibit a significantly higher prevalence among food isolates, the environment and animal clinical cases (9). Lineage III and IV strains account for approximately 1% of human listeriosis cases in humans but are more prominent in animals (24). Most sporadic human listeriosis cases appear to be caused by serotype-4b and -1/2a strains, while most human listeriosis outbreaks have been linked to serotype-4b strains (11). Outbreaks have rarely occurred because of non-4b serotypes but do happen (8)). For example, a serotype-1/2a outbreak of gastrointestinal listeriosis was linked to sliced turkey in the United States (8).

In the United States, there is a zero-tolerance policy for *L. monocytogenes* in food that requires the recall of any adulterated food product (18). *L. monocytogenes* has been investigated in various food products such as seafood, dairy products, meats, and RTE products. In the past, there were listeriosis outbreaks in the United States linked to contaminated cantaloupes, soft cheeses, RTE turkey deli meat, ice cream, unpasteurized milk, candied apples, packages and frozen vegetables, and most recently, soft raw milk cheeses (25).

There has been limited focus on *Listeria* spp. related to poultry and poultry products. To date, there have been no poultry (chickens, specifically)-related listeriosis outbreaks; however, though uncommon, poultry flocks can be contaminated with *L. monocytogenes* and result in sporadic listeriosis cases (3, 5, 26). In the few studied cases where the disease was attributed to poultry sources, symptoms have included septicemia or localized encephalitis (5). Poultry flocks can serve as a reservoir and can contaminate the litter and surrounding environments (3, 5, 27–29). Although rare cases of human listeriosis from raw poultry meat has stemmed from contamination and unhygienic practices of processing environments (4, 5), little is known about the prevalence within poultry and poultry products. Therefore, the purpose of the remainder of this review is to take a farm-to-fork approach and highlight the possible issues and places of potential *Listeria* spp. contact during the production of poultry and the importance for continued research of *Listeria* spp. in poultry to reduce the potential of future outbreaks.

## Live Poultry Production Practices

Consumers have repeatedly expressed concerns about animal welfare related to intensive chicken farming. They want to be sure that all animals being raised for food are treated with respect and are properly cared for during their lives. Farmers and companies share the public’s concern and recognize that they have an ethical obligation to make sure that the animals on their farms are well cared for (30). Guidelines for poultry management include hatchery operations, appropriate housing and space, proper nutrition and feeding, health care, and monitoring, and these guidelines are provided within handbooks to poultry breeders (31), industry association protocols (30), or legislative texts (32).

Housing type and management are dictated by the type of poultry being produced (broiler versus layers), economics and the preferences in a particular region and climate. Globally, poultry housing must provide comfortable and protective shelter for the birds and effective measures must be established to protect flock health and minimize any negative impact on bird welfare (30). Several housing systems are used including conventional cages, furnished/enriched cages, barn systems, or free-range systems which exist for layers (33). The most common housing systems around the world remain cages with a space allowance ranging from 300 to 750 cm^2^ according to the legislation of the country (34). Broilers are generally held in groups in environmentally controlled housing, open and naturally ventilated poultry houses or on free range (34). In grow-out houses, the minimum space should be one-half square foot per bird as stated by the Council for Agricultural Science and Technology (CAST) (30). Whatever the rearing system, poultry housing and equipment must be designed to protect the birds from environmental conditions. Appropriate ventilation and heater systems are needed to regulate seasonal temperatures to keep air moving throughout the house and to provide optimal air quality at any time (30, 31). The bedding material must be of good quality and the litter must be kept clean. Ammonia emissions must be monitored and appropriate measures be taken, if necessary to reduce to the minimum allowable level by appropriate measures, if necessary (30, 31, 35).

Nutrition and water requirements for poultry depend on a range of factors including the commercial goals of the poultry enterprise, type of bird, breed and age, or stage of development (30, 31, 36). A good-quality dietary feed will help the birds stay healthy and grow well. Birds should have unlimited access to clean, fresh, and good quality drinking water. All feeding and drinking systems must be checked for proper operation daily and must be adjusted in height as the birds grow. Precise and complete guidelines about the nutrition and water requirements, as well as the feeding and drinking systems, are given by poultry breeders (31). A good poultry health management is an important component of flock management and meat or egg production (37). Good hygienic conditions within the poultry house must be achieved through the implementation of correct biosecurity, cleaning, and vaccination programs. A working relationship with an avian veterinarian is an integral part of health management (30, 31).

## *Listeria* spp. within the Hatchery Environment

Live poultry production includes the hatchery and the grow-out farm environments. These two steps of poultry production may contribute to the contamination of live birds with *L. monocytogenes* and potentially lead to the contamination in the food processing plant and the poultry meat. The hatchery is responsible for the incubation of fertile eggs obtained from parent breeders and the hatching of chicks. The hatchery is the first production stage where eggshell surfaces, embryos, and chicks can be contaminated by pathogenic bacteria. Very few studies have investigated the occurrence of *Listeria* spp. and *L. monocytogenes* in the hatchery environment, Over 200 samples collected in three commercial broiler hatcheries in northern Georgia, USA, only 1% of chick paper pads and 6% of eggshell fragments were positive for *L. monocytogenes* (38). In Thailand, 32 hatcheries were inspected for *L. monocytogenes* over a 5-year period. Incubator trays of equipment used in hatcheries were swabbed (548 samples) and meconium from 10-day-old chicks were collected (523 samples). *L. monocytogenes* was not detected in 1,071 samples over this 5-year period (39).

Overall, limited data are available on the occurrence of *Listeria* spp. and *L. monocytogenes* in the hatchery environment. The vertical transmission of *L. monocytogenes* from the parent flock to the day-old chicken leaving the hatchery has not fully been investigated, contrary to other foodborne pathogens such as *Salmonella* (40, 41) and *Campylobacter* (42, 43). Since *L. monocytogenes* contamination of poultry products has focused on the processing stage (44, 45) and RTE products (1, 6), the hatchery environment has not been viewed as important to poultry meat contamination processes. However, there is no evidence to exclude the hatchery environment in the early contamination of chicks and consequently the final product.

## *Listeria* spp. within the Grow-Out Farm Environment

Once the 1-day-old chicks leave the hatchery environment, they are shipped to the grow-out farms to reach pre-determined size/weight based on the processing/final product requirements. Studies reporting the prevalence of *Listeria* spp. and *L. monocytogenes* in the grow-out farm environment vary according to the country, the number of farms and flocks examined, the breed of bird, and the type of samples collected (Table 1). To evaluate the contamination rate of *Listeria* spp. and *L. monocytogenes* on grow-out farms, studies have either investigated its prevalence in environmental samples surrounding or within the production area (soil, grass, dust, litter, feed, drinking water, layer egg shells, nest boxes) or in bird ceca and feces. In studies focusing on environmental samples, *Listeria* spp. prevalence ranged from as low as 1.4 to 53% (5, 6, 46–49). According to the sample type, 9.8 to 52.5, 70, 10, 30, and 6 to 42.8% of the samples were positive for *Listeria* spp. in broiler litter, farm feed, farm drinking water, soil, and grass samples, respectively (5, 46–48). Milillo et al. (46) demonstrated that environmental samples collected from the pasture before broiler introduction were rarely positive for *Listeria* spp. (5%), whereas samples collected after broiler exposure were significantly more likely to contain *Listeria* spp. (53%) (46). These findings implicate poultry as a source of the *Listeria* spp. being found in these environmental samples. Not only *Listeria* spp. have been isolated from environmental samples from grow-out farms but also they have also been isolated directly from the poultry. An investigation in Danish broilers and the broiler house environment of 71 flocks. *Listeria* spp. was identified in 9.8% of litter samples and 17% of fecal samples yielding an overall prevalence of 14% (10/71) in broiler flocks (47). A lower prevalence of *Listeria* spp. (4.7%) was found in 150 fresh fecal droppings collected at four chicken farms in the suburbs of Tokyo (50). *Listeria* spp. were more likely to be isolated from young broilers, suggesting that as the birds’ intestinal microbiota develop, their levels of *Listeria* spp. decline (46). *L. innocua* is the predominant species found on grow-out farms, representing ≤78% of all isolated *Listeria* spp. (5, 46, 47, 50, 51). *L. innocua* is important because it is closely related to *L. monocytogenes* and both are genetically similar (6, 52). Other *Listeria* species, such as *L. ivanovii, L. welshimeri*, and *L. seeligeri*, have also been identified in environmental farm samples or chicken feces but their detection remain infrequent (5, 50).

**Table 1:** Prevalence of *L. monocytogenes* during live production in grow-out farm environments.

Country	Number of farms or flocks involved		Sample collected	*L. monocytogenes* prevalence (%)			*L. monocytogenes* lineages/serovars	Reference
				Per sample	Per flock			
Denmark	71 broiler flocks		Wet litter Feces	1.5% (1/67) 2.1% (1/48)	3% (2/71)		ND	(47)
Denmark	236 parents, 5 flocks		Cecal content	4.7% (11/236)	ND		ND	(53)
	2078 broilers, 90 flocks			0% (0/2,078)	ND			
Egypt	20 farms, 200 samples		Litter Poultry feed Drinking water	2.5% (2/80) 0% (0/20) 0% (0/20)	ND		ND	(54)
France	84 cage-layer flocks		Feces Dust	28.6% (total)	30.9% (26/84)		ND	(55)
	142 broiler chicken flocks		Boot swabs	46.2%	31.7% (45/142)			
France	200 laying hen flocks	88 caged-flocks	Feces Dust	10.5% (45/429) 5% (7/139)	29.5% (26/88)	15.5% (31/200)	1/2a, 1/2b and 4e, 4b	(56)
		112 floor-reared flocks	Dust	2.9% (6/206)	4.5% (5/112)		1/2a	
	145 broiler flocks	85 conventional flocks	Boot swabs	12.7% (54/425)	28.2% (24/85)	31.7% (46/145)	1/2a, 1/2b and 4e, 4b	
		60 free-range flocks		36.7% (22/60)	36.7% (22/60)		1/2a, 1/2b and 4e, 4b	
France	75 breeding turkey flocks		Feces	4.8% (18/375)	12% (9/75)		1/2a	(57)
	86 fattening turkey flocks			2.6% (11/428)	9.3% (8/86)		1/2a, 4b	
Japan	4 chicken farms		Feces	0% (0/150)	ND		ND	(50)
Spain	60 free-range chicken flocks		Feces	ND	26.7% (16/60)		4b or 4e	(58)
Thailand	43 breeder farms 1,331 broiler farms		Litter Poultry feed Drinking water	0% (0/2,504) 0% (0/2,215) 0% (0/2,398)	ND		ND	(39)
USA	340 broilers 280 layers		Grass Soil Ceca	4.8% (1/21) 0% (0/20) 1% (4/399)	Lineage II (1/2a, 1/2c, 3a) Lineage III (4a, 4b, 4c)		ND	(6, 46)
United Kingdom	Local farm		Litter Hens feces Duck feces	11.1% (1/9) 20% (1/5) 16.7% (1/6)	ND		ND	(51)

*ND, not determined*.

*Listeria monocytogenes* has been isolated on grow-out farms from litter (51, 59), dust (56), grass (46), feed (60), feces (56, 61, 62), and cecal (46, 53) samples, with an overall contamination rate ranging from 0 to 46.2% in those samples. The prevalence of *L. monocytogenes* in poultry ceca and feces is generally low but can be highly variable, ranging from 0 to 32% (39, 47, 50, 51, 53, 56). Moreover, only a small percentage of birds may be long-term carriers of the organism (63). Intestinal carriage of *L. monocytogenes* may be transient, most likely resulting from ingestion of *Listeria*-contaminated feed, soil, and/or drinking water. Indeed, it has rarely been proven that poultry feed and drinking water can be contaminated with *Listeria* spp. generally (54) or *L. monocytogenes* specifically (60, 64). Broiler flock contamination rates for *L. monocytogenes* can range from 3 to 32% (47, 55, 56, 58), with similar contamination rates for layer hens and turkey flocks (55–57). The number of positive samples within positive flocks is generally low, with most of the positive flocks (32 to 55.6%) represented by only one positive sample (55–57). These results suggest that poultry may not represent a common reservoir for *L. monocytogenes* in the grow-out farm environment, although variability in its prevalence among broiler flocks makes poultry a potential source of *L. monocytogenes* that should be further investigated.

Poultry can shed *Listeria* spp. and *L. monocytogenes* in fecal material and contaminate elements of the grow-out farm environment, including the poultry house where studies have shown that ≤52.5 and ≤25% of litter samples were positive for *Listeria* spp. and *L. monocytogenes*, respectively (51, 54, 59). Chemaly et al. (56) showed that *L. monocytogenes* detection within samples collected from caged laying hen flocks was dependent on sample type. When only *L. monocytogenes*-positive flocks were considered, the difference between dust and fecal samples were strongly significant, with a greater detection in feces than in dust samples (56). This may be attributed to dust samples being contaminated by contact with fecal materials shed on the floor. A potential transfer of *Listeria* spp. between broilers and their environment has been shown by Milillo et al. (46). The circulation of *Listeria* spp., especially *L. monocytogenes*, between animals and farm environment has also been observed in ruminant farming systems (65, 66).

Poultry farm characteristics and management practices also have an influence on the prevalence of *L. monocytogenes* in bird flocks. These risk factors are mostly related to the hygienic status of the house and sanitary measures applied to the flocks. Aury et al. (55) identified six risk factors significantly associated with *L. monocytogenes* contamination at the end of the broiler flocks production cycle (55). The risk of *L. monocytogenes* contamination was increased when (i) farmers did not respect the principle of two areas (clean and dirty) at the poultry house entrance, (ii) disinfection was not carried out between flocks by spraying, (iii) there was an absence of pest control of the poultry house before the arrival of the next flock, (iv) litter was not protected during storage, (v) farm staff cared for other broiler houses, and (vi) the watering system did not consist of nipples with cups. Within the same study, three risk factors significantly increased *L. monocytogenes* contamination in caged hen flocks at the end of the laying period: (i) presence of pets in the production site, (ii) type of feed (use of meal rather than crumbs/crumbles), and (iii) insufficient or incomplete removal of fecal droppings (e.g., conveyor belt with deep pit storage or deep pit only methods not used) (55).

The prevalence of *L. monocytogenes* contamination may also be dependent of the type of production system. No significant differences in the prevalence of *Listeria* spp. were found on and within eggs and in the environment of a sister flock of conventional cage and free-range laying hens. In this study, *L. monocytogenes* represented 28.5% (2/7) of the *Listeria* spp. isolated (48). This result is supported by Schwaiger et al. (49) who compared cloacal swabs from 20 conventional and organic egg farms in Germany and found no significant difference in *Listeria* spp. between production methods (49). Conversely, Chemaly et al. (56) showed a significant difference between caged- and floor-reared hens with a greater detection of *L. monocytogenes* in dust samples from floor-reared hens, in *L. monocytogenes*-positive flocks (56). Alternatively raised broilers (e.g., all natural, pastured) represent management systems with unknown food safety implications, considering these poultry are raised in less controlled environments than conventionally raised birds (67). Poultry farms frequently have other animals (beef cattle, sheep, goats, or swine) and pets present on the production site (67). These animals can be reservoirs for and play a role in the multiplication and excretion of *L. monocytogenes* into the environment. The presence of other animals during grow-out was found to increase the risk of *L. monocytogenes* contamination in laying hen flocks (55). Because *L. monocytogenes* is commonly associated with other farm animals and is a natural saprophyte, the occurrence of *L. monocytogenes* in alternatively raised poultry is of particular interest.

Few studies have identified *L. monocytogenes* from poultry farms at the serogroup level. From these, serovar 1/2a represented a dominant proportion of the isolates regardless of the bird species (46, 56, 57). To a lesser extent, serovars 1/2b and 4e/4b were also identified in laying hen, broiler and turkey flocks (56–58). Although serovar 1/2a did not differ between caged- and floor-reared hens, or between standard and free-range systems for broilers, serovars 1/2b and 4e/4b were significantly more prevalent in broilers (56). Although rarer in poultry than other important foodborne pathogens (*Salmonella, Campylobacter*), the above discussions show that *Listeria* spp., and specifically *L. monocytogenes*, can be present both within the birds (ceca and feces), and the grow-out farm environment. These data highlight the potential for the live birds to be a vector for this pathogen to enter the processing environment, which is the side most commonly viewed as the greatest risk for *L. monocytogenes* contamination.

## Potential for Listeric Infections in Poultry

Although poultry can be an asymptomatic carrier of *L. monocytogenes*, they can also develop, in rare cases, listeric infections (27, 62). Only a few sporadic clinical outbreaks have been described. An outbreak of listeriosis was reported in a backyard poultry flock in Washington State was attributed to serotype 4b the source of the infection (26). *L. monocytogenes* serovar 4b was also involved in an outbreak of listeriosis in a pheasant breeder farm of Jingzhou, Hubei Province, China (68). There is greater evidence for potential systemic *Listeria* infection in other avian species, such as turkeys. Huff et al. (69) demonstrated in young turkey poults inoculated with a high or low dose of *L. monocytogenes* Scott A in the air sac that the high dosed birds reached 100% mortalities in 2 weeks, and the *Listeria* challenge strain could be isolated from the liver, pericardium, brain, both knee joints, suggesting that *L. monocytogenes* Scott A could be invasive through the respiratory system of susceptible turkey poults. In a follow-up challenge study comparing oral or oculonasal routes, Huff et al. (70) demonstrated that the oculonasal route led to greater mortalities and lower body weights than orally challenged birds. Stress may be a factor as well. When Huff et al. (71) exposed 13-week-old male turkeys to an immunosuppressive treatment and stress associated with transport, they observed an increase *Listeria* colonization in older birds. There are no comparable studies conducted with commercial poultry but a recent study by Jarvis et al. (72) demonstrated that *L. monocytogenes* strains could infect HD11 chicken macrophage-like cells and that infection leads to an initial halt in growth of the HD11 cells for at least 11 h before the HD11 cells begin to lyse. The authors used this as evidence to suggest there could be sufficient time for *Listeria* infected macrophages to circulate in the blood and potentially infect other tissues in the chicken. Therefore, it would be interesting to compare cellular mechanisms of these *Listeria* infected HD11 cells with other avian species such as turkeys as well non-avian macrophages such as those from mice.

## Conclusion

*Listeria* is considered one of the major bacterial foodborne pathogens but it is often not considered epidemiologically important in poultry production, although there is nothing about the poultry production and processing environments that preclude the survival and persistence of *Listeria* spp. While sporadic and very isolated cases of listeriosis have been attributed to poultry, *Listeria* spp., and specifically *L. monocytogenes* have been isolated from all stages of the poultry production and processing continuum. Grow-out farm studies show that live birds are an important potential vector for *Listeria* contamination of the processing environment. Different studies have described factors related to the survival of *Listeria* within processing facilities, but there is a paucity of evidence linking live production and processing environments. The following food safety-related question must be asked: Does *Listeria* contamination of poultry meat come from poultry or its environment? To address this question, there is a need to better understand the genetics of *Listeria* spp. and *L. monocytogenes* isolated from poultry environments as compared to other sources and listeriosis outbreaks attributed to poultry. If the epidemiological and genetic factors related to *Listeria* prevalence and pathogenicity can be elucidated, there is an opportunity to better assess the potential public health effects of *Listeria* from the poultry industry and develop management practices or treatments to mitigate these effects.

## Author Contributions

The authors declare that there is no conflict of interest and contribution was equally distributed among authors.

## Conflict of Interest Statement

The authors declare that the research was conducted in the absence of any commercial or financial relationships that could be construed as a potential conflict of interest. The handling editor declared a shared affiliation, though no other collaboration, with several of the authors MR, AL, and TM and states that the process nevertheless met the standards of a fair and objective review.
